# The dicyclo­hexyl­amine salt of RG108 (*N*-phthalyl-l-tryptophan), a potential epigenetic modulator

**DOI:** 10.1107/S160053681004626X

**Published:** 2010-11-13

**Authors:** Julie Braun, Irving Boittiaux, Anaelle Tilborg, Didier Lambert, Johan Wouters

**Affiliations:** a Department of Chemistry, University of Namur, 61, Rue de Bruxelles, B-5000 Namur, Belgium; b Louvain Drug Research Institute (LDRI), UCL, 50, Avenue Mounier, B-1200 Woluwe-Saint-Lambert, Belgium

## Abstract

The dicyclo­hexyl­amine salt of RG108 (*N*-phthalyl-l-tryptophan) co-crystallizes with a water mol­ecule and a disordered mol­ecule of dimethyl­formamide (DMF), *viz*. dicyclo­hexyl­aminium (*S*)-2-(1,3-dioxoisoindolin-2-yl)-3-(1*H*-indol-3-yl)propanoate dimethyl­formamide solvate monohydrate, C_12_H_24_N^+^·C_19_H_13_N_2_O_4_^−^·C_3_H_7_NO·H_2_O. The conformation of the deprotonated compound is constrained by charge-assisted strong hydrogen bonds with the dicyclo­hexyl­aminium ion and a dense hydrogen-bond network involving co-crystallized solvent mol­ecules. The dihedral angle between the fused ring systems in the anion is 58.35 (4)°.

## Related literature

For the synthesis and biological evaluation, see: Brueckner *et al.* (2005 ▶).
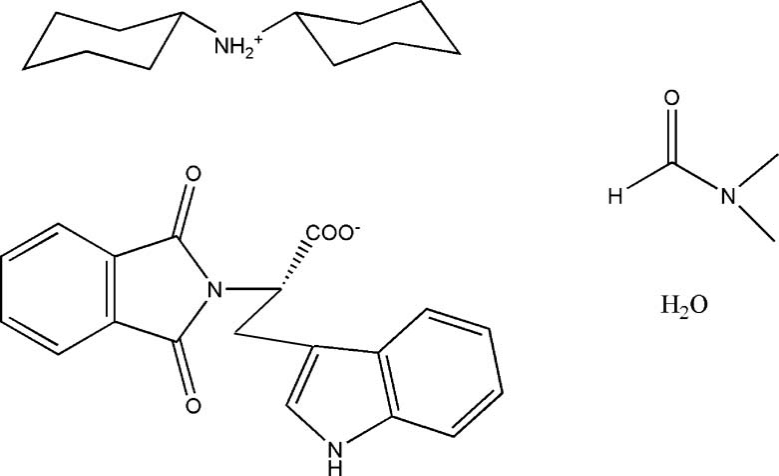


## Experimental

### 

#### Crystal data

C_12_H_24_N^+^·C_19_H_13_N_2_O_4_^−^·C_3_H_7_NO·H_2_O
*M* *_r_* = 606.75
Orthorhombic, 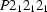

*a* = 9.0884 (1) Å
*b* = 15.0206 (3) Å
*c* = 24.4749 (5) Å
*V* = 3341.15 (10) Å^3^
*Z* = 4
Cu *K*α radiation
μ = 0.67 mm^−1^
*T* = 293 K
0.55 × 0.04 × 0.03 mm

#### Data collection

Oxford Diffraction Xcalibur diffractometer with a Ruby (Gemini ultra Cu) detector
Absorption correction: multi-scan (*CrysAlis PRO*; Oxford Diffraction, 2009 ▶) *T*_min_ = 0.967, *T*_max_ = 0.981
13362 measured reflections
5552 independent reflections
4936 reflections with *I* > 2σ(*I*)
*R*_int_ = 0.026

#### Refinement

*R*[*F* ^2^ > 2σ(*F* ^2^)] = 0.037
*wR*(*F* ^2^) = 0.094
*S* = 1.01
5552 reflections
409 parameters
8 restraints
H atoms treated by a mixture of independent and constrained refinement
Δρ_max_ = 0.30 e Å^−3^
Δρ_min_ = −0.27 e Å^−3^
Absolute structure: Flack (1983 ▶), 2160 Friedel pairs
Flack parameter: 0.02 (18)

### 

Data collection: *CrysAlis PRO* (Oxford Diffraction, 2009 ▶); cell refinement: *CrysAlis PRO*; data reduction: *CrysAlis PRO*; program(s) used to solve structure: *SHELXS97* (Sheldrick, 2008 ▶); program(s) used to refine structure: *SHELXL97* (Sheldrick, 2008 ▶); molecular graphics: *ORTEPIII* (Burnett & Johnson, 1996 ▶) and *PLATON* (Spek, 2009 ▶); software used to prepare material for publication: *SHELXL97*.

## Supplementary Material

Crystal structure: contains datablocks I, global. DOI: 10.1107/S160053681004626X/vm2050sup1.cif

Structure factors: contains datablocks I. DOI: 10.1107/S160053681004626X/vm2050Isup2.hkl

Additional supplementary materials:  crystallographic information; 3D view; checkCIF report

## Figures and Tables

**Table 1:** Hydrogen-bond geometry (Å, °)

*D*—H⋯*A*	*D*—H	H⋯*A*	*D*⋯*A*	*D*—H⋯*A*
N3—H*N*3*B*⋯O4^i^	0.86 (3)	1.88 (3)	2.7309 (19)	169 (2)
N2—H2⋯O5^ii^	0.86	1.97	2.813 (3)	165
N3—H*N*3*A*⋯O3^iii^	0.99 (3)	1.79 (3)	2.7740 (19)	173 (2)
O5—H5*B*⋯O99	0.83 (4)	1.84 (4)	2.645 (4)	164 (4)
O5—H5*C*⋯O1^iv^	0.89 (4)	1.99 (4)	2.857 (2)	164 (3)

Symmetry codes: (i) 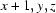; (ii) 
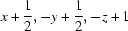
; (iii) 
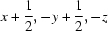
; (iv) 
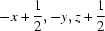
.

**Fig. 1.**
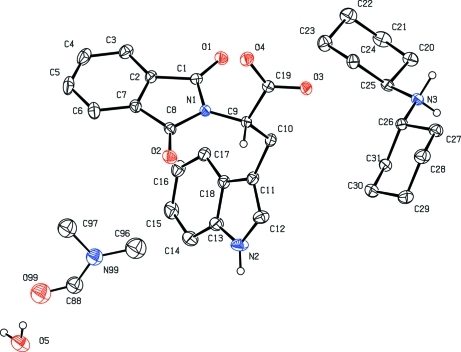
ORTEP view (with atom numbering) of the title compound. Only selected H atoms have been retained for clarity (on the chiral carbon, on the protonated nitrogen, and H involved in H-bonds). Displacement ellipsoids for non-H atoms are drawn at the 30% probability level.

## supplementary crystallographic information

### Comment

RG108 (N-phthalyl-L-tryptophan) is a DNA methyltransferase (DMNT) inhibitor that was discovered by virtual screening (Brueckner *et al.*, 2005). It reactivates tumor suppressor gene expression in tumor cells by DNA demethylation. RG108 also inhibits human tumor cell line proliferation.

Isomer *S* (C9) of RG108 is obtained starting from L-tryptophan and phthalic anhydride in DMF.

The unprotonated carboxylate group (both C—O bond lenghts are similar with C19—O3 = 1.246 (2) Å and C19—O4 = 1.247 (2) Å) of RG108 is close to the protonated *sp^3^* nitrogen atom (N3) of the amine (intermolecular O···N distances: O3···N3*^i^* = 2.774 (2) Å and O4···N3*^ii^* = 2.731 (2) Å; *i* = -1/2 + *x*,1/2 - *y*,-*z*, *ii* = -1 + *x*,*y*,*z*, see also Table 1).

A water molecule (O5) has co-crystallized and is involved in the stability of the packing as it forms a network of H-bonds connecting the N—H (N2) of the indole ring of RG108 with a carbonyl function (O1) of the phtalimide ring of a symmetry-related molecule and the oxygen atom (O99) of a molecule of DMF solvent (Table 1).

In addition to H-bonding to the water, the extra (disordered) co-crystallized solvent molecule of DMF is thightly packed in a cavity formed by the aromatic heterocycles of RG108 (the phtalimide and the indole rings).

As a consequence of the dense packing (salt bridge, H-bonds and van der Waals interactions), the two aromatic, planar, heterocycles of RG108 are perpendicular (acute angle between the planes defined by the phtalimide and the indole rings = 58.35 (4)°).

### Experimental

Synthesis of the compound was made by micro-ave heating of L-tryptophane and phthalic anhydride in DMF by adapting the procedure described by Brueckner *et al.* (2005).

Crystals were obtained by evaporation at room temperature of a solution in mixture of methylene chloride and methanol (9/1).

### Refinement

The two H atoms of the water molecule and the two H atoms on (protonated) nitrogen N5 were located from ΔF Fourier difference maps and their position refined. All other H atoms were placed at idealized positions and allowed to ride on their parent atoms.

Atoms of a DMF molecule were refined isotropically. Disorder has been taken into account by refining two sets of coordinates (0.7 and 0.3 occupancies respectively) for each atom of the DMF molecule. Bond lengths and valence angles were restrained to be similar in both disordered parts.

### Crystal data

**Table:**

C_12_H_24_N^+^·C_19_H_13_N_2_O_4_^−^·C_3_H_7_NO·H_2_O	*F*(000) = 1304.0
*M**_r_* = 606.75	*D*_x_ = 1.206 Mg m^−^^3^
Orthorhombic, *P*2_1_2_1_2_1_	Cu *K*α radiation, λ = 1.54184 Å
Hall symbol: P 2ac 2ab	Cell parameters from 7270 reflections
*a* = 9.0884 (1) Å	θ = 3.5–67.3°
*b* = 15.0206 (3) Å	µ = 0.67 mm^−^^1^
*c* = 24.4749 (5) Å	*T* = 293 K
*V* = 3341.15 (10) Å^3^	Needle, yellow
*Z* = 4	0.55 × 0.04 × 0.03 mm

### Data collection

**Table:**

Oxford Diffraction Xcalibur diffractometer with a Ruby (Gemini ultra Cu) detector	5552 independent reflections
Radiation source: Enhance Ultra (Cu) X-ray Source	4936 reflections with *I* > 2σ(*I*)
mirror	*R*_int_ = 0.026
Detector resolution: 10.3712 pixels mm^-1^	θ_max_ = 67.4°, θ_min_ = 3.5°
ω scans	*h* = −10→9
Absorption correction: multi-scan (*CrysAlis PRO*; Oxford Diffraction, 2009)	*k* = −15→17
*T*_min_ = 0.967, *T*_max_ = 0.981	*l* = −28→29
13362 measured reflections	

### Refinement

**Table:**

Refinement on *F*^2^	Secondary atom site location: difference Fourier map
Least-squares matrix: full	Hydrogen site location: inferred from neighbouring sites
*R*[*F*^2^ > 2σ(*F*^2^)] = 0.037	H atoms treated by a mixture of independent and constrained refinement
*wR*(*F*^2^) = 0.094	[1.00000 + 0.00000exp(0.00(sinθ/λ)^2^)]/ [σ^2^(*F*_o_^2^) + 0.0000 + 0.0000**P* + (0.0611*P*)^2^ + 0.0400sinθ/λ] where *P* = 0.33333*F*_o_^2^ + 0.66667*F*_c_^2^
*S* = 1.01	(Δ/σ)_max_ < 0.001
5552 reflections	Δρ_max_ = 0.30 e Å^−^^3^
409 parameters	Δρ_min_ = −0.27 e Å^−^^3^
8 restraints	Absolute structure: Flack (1983), 2160 Friedel pairs
Primary atom site location: structure-invariant direct methods	Flack parameter: 0.02 (18)

### Special details

**Table:**

Geometry. All e.s.d.'s (except the e.s.d. in the dihedral angle between two l.s. planes) are estimated using the full covariance matrix. The cell e.s.d.'s are taken into account individually in the estimation of e.s.d.'s in distances, angles and torsion angles; correlations between e.s.d.'s in cell parameters are only used when they are defined by crystal symmetry. An approximate (isotropic) treatment of cell e.s.d.'s is used for estimating e.s.d.'s involving l.s. planes.
Refinement. Refinement of *F*^2^ against ALL reflections. The weighted *R*-factor *wR* and goodness of fit *S* are based on *F*^2^, conventional *R*-factors *R* are based on *F*, with *F* set to zero for negative *F*^2^. The threshold expression of *F*^2^ > σ(*F*^2^) is used only for calculating *R*-factors(gt) *etc*. and is not relevant to the choice of reflections for refinement. *R*-factors based on *F*^2^ are statistically about twice as large as those based on *F*, and *R*- factors based on ALL data will be even larger.

### Fractional atomic coordinates and isotropic or equivalent isotropic displacement parameters (Å^2^)

**Table:**

	*x*	*y*	*z*	*U*_iso_*/*U*_eq_	Occ. (<1)
C1	0.28198 (19)	0.08359 (12)	0.15901 (7)	0.0255 (4)	
C2	0.15124 (19)	0.04587 (13)	0.18771 (7)	0.0281 (4)	
C3	0.0899 (2)	−0.03769 (15)	0.18579 (8)	0.0371 (4)	
H3	0.1306	−0.0828	0.1646	0.044*	
C4	−0.0364 (2)	−0.05175 (17)	0.21717 (10)	0.0471 (6)	
H4	−0.0803	−0.1077	0.2171	0.056*	
C5	−0.0976 (2)	0.01558 (18)	0.24826 (9)	0.0466 (6)	
H5	−0.1820	0.0041	0.2686	0.056*	
C6	−0.0356 (2)	0.09991 (17)	0.24977 (9)	0.0409 (5)	
H6	−0.0774	0.1455	0.2703	0.049*	
C7	0.09121 (18)	0.11355 (14)	0.21952 (7)	0.0293 (4)	
C8	0.18476 (18)	0.19374 (14)	0.21386 (7)	0.0276 (4)	
C9	0.40737 (17)	0.23259 (12)	0.15742 (7)	0.0245 (4)	
H9	0.3868	0.2900	0.1749	0.029*	
C10	0.56294 (17)	0.20499 (14)	0.17540 (7)	0.0283 (4)	
H10A	0.5840	0.1463	0.1609	0.034*	
H10B	0.6334	0.2461	0.1596	0.034*	
C11	0.58403 (18)	0.20322 (13)	0.23613 (8)	0.0300 (4)	
C12	0.6295 (2)	0.27212 (15)	0.26820 (8)	0.0382 (5)	
H12	0.6514	0.3289	0.2553	0.046*	
C13	0.5969 (2)	0.15904 (15)	0.32564 (8)	0.0365 (4)	
C14	0.5908 (2)	0.10309 (17)	0.37118 (9)	0.0444 (5)	
H14	0.6146	0.1238	0.4059	0.053*	
C15	0.5484 (2)	0.01611 (17)	0.36273 (9)	0.0464 (6)	
H15	0.5429	−0.0226	0.3923	0.056*	
C16	0.5136 (2)	−0.01489 (15)	0.31049 (9)	0.0407 (5)	
H16	0.4854	−0.0739	0.3059	0.049*	
C17	0.52024 (19)	0.04037 (14)	0.26543 (8)	0.0329 (4)	
H17	0.4971	0.0188	0.2309	0.039*	
C18	0.56222 (18)	0.12900 (14)	0.27259 (7)	0.0297 (4)	
C19	0.39345 (18)	0.24740 (12)	0.09508 (7)	0.0251 (4)	
C20	1.0315 (2)	0.06650 (15)	0.02751 (8)	0.0362 (4)	
H20A	1.1333	0.0726	0.0389	0.043*	
H20B	1.0264	0.0797	−0.0112	0.043*	
C21	0.9818 (3)	−0.02937 (15)	0.03696 (10)	0.0466 (5)	
H21A	1.0377	−0.0688	0.0134	0.056*	
H21B	1.0017	−0.0460	0.0745	0.056*	
C22	0.8190 (3)	−0.04061 (16)	0.02532 (10)	0.0460 (5)	
H22A	0.7894	−0.1011	0.0338	0.055*	
H22B	0.8004	−0.0301	−0.0132	0.055*	
C23	0.7290 (2)	0.02426 (15)	0.05937 (9)	0.0417 (5)	
H23A	0.6254	0.0169	0.0510	0.050*	
H23B	0.7431	0.0114	0.0979	0.050*	
C24	0.7748 (2)	0.11973 (14)	0.04766 (8)	0.0325 (4)	
H24A	0.7539	0.1338	0.0098	0.039*	
H24B	0.7179	0.1598	0.0704	0.039*	
C25	0.93764 (19)	0.13371 (13)	0.05872 (7)	0.0277 (4)	
H25	0.9559	0.1267	0.0979	0.033*	
C26	0.90346 (18)	0.30325 (13)	0.06517 (7)	0.0265 (4)	
H26	0.7980	0.2957	0.0582	0.032*	
C27	0.9557 (2)	0.38741 (14)	0.03688 (8)	0.0350 (4)	
H27A	0.9352	0.3831	−0.0019	0.042*	
H27B	1.0613	0.3931	0.0414	0.042*	
C28	0.8803 (3)	0.46978 (15)	0.06000 (9)	0.0430 (5)	
H28A	0.9198	0.5225	0.0425	0.052*	
H28B	0.7758	0.4671	0.0521	0.052*	
C29	0.9029 (2)	0.47659 (15)	0.12145 (9)	0.0424 (5)	
H29A	1.0063	0.4862	0.1293	0.051*	
H29B	0.8480	0.5270	0.1356	0.051*	
C30	0.8515 (2)	0.39192 (16)	0.14926 (9)	0.0409 (5)	
H30A	0.7461	0.3857	0.1443	0.049*	
H30B	0.8706	0.3963	0.1882	0.049*	
C31	0.92799 (19)	0.30959 (14)	0.12677 (7)	0.0317 (4)	
H31A	0.8892	0.2568	0.1445	0.038*	
H31B	1.0326	0.3129	0.1344	0.038*	
N1	0.29621 (15)	0.17046 (10)	0.17706 (6)	0.0251 (3)	
N2	0.6388 (2)	0.24668 (12)	0.32209 (7)	0.0429 (4)	
H2	0.6661	0.2799	0.3489	0.051*	
N3	0.98457 (16)	0.22521 (11)	0.04155 (6)	0.0269 (3)	
O1	0.36330 (14)	0.04770 (9)	0.12646 (6)	0.0342 (3)	
O2	0.17295 (15)	0.26576 (10)	0.23543 (6)	0.0374 (3)	
O3	0.50423 (13)	0.27765 (10)	0.07154 (5)	0.0331 (3)	
O4	0.27256 (13)	0.23090 (11)	0.07320 (6)	0.0383 (3)	
O5	0.1772 (2)	0.13211 (12)	0.59148 (8)	0.0539 (4)	
O99	0.0737 (4)	0.1983 (3)	0.49899 (14)	0.0750 (9)*	0.70
N99	0.1552 (5)	0.2310 (3)	0.41554 (16)	0.0510 (11)*	0.70
C88	0.1620 (4)	0.2142 (3)	0.46712 (15)	0.0517 (8)*	0.70
H88	0.2571	0.2153	0.4812	0.062*	0.70
C96	0.2644 (6)	0.2521 (4)	0.3781 (2)	0.0796 (13)*	0.70
H96A	0.2211	0.2610	0.3428	0.119*	0.70
H96B	0.3342	0.2042	0.3762	0.119*	0.70
H96C	0.3136	0.3056	0.3894	0.119*	0.70
C97	−0.0037 (4)	0.2278 (3)	0.39149 (17)	0.0656 (10)*	0.70
H97A	−0.0004	0.2411	0.3531	0.098*	0.70
H97B	−0.0642	0.2709	0.4098	0.098*	0.70
H97C	−0.0443	0.1694	0.3968	0.098*	0.70
O99B	0.0109 (6)	0.2045 (4)	0.5007 (2)	0.0357 (11)*	0.30
N99B	0.1149 (8)	0.2235 (5)	0.4188 (3)	0.0317 (18)*	0.30
C88B	0.0171 (12)	0.2275 (8)	0.4510 (4)	0.071 (3)*	0.30
H88B	−0.0696	0.2516	0.4373	0.085*	0.30
C96B	0.2691 (11)	0.2064 (7)	0.4520 (4)	0.067 (2)*	0.30
H96D	0.3491	0.2029	0.4265	0.100*	0.30
H96E	0.2624	0.1517	0.4721	0.100*	0.30
H96F	0.2860	0.2548	0.4769	0.100*	0.30
C97B	0.1649 (19)	0.2484 (12)	0.3692 (7)	0.112 (5)*	0.30
H97D	0.2612	0.2238	0.3633	0.168*	0.30
H97E	0.1701	0.3122	0.3674	0.168*	0.30
H97F	0.0992	0.2270	0.3414	0.168*	0.30
H5B	0.133 (4)	0.145 (3)	0.5631 (15)	0.072 (11)*	
H5C	0.150 (4)	0.076 (3)	0.5979 (13)	0.072 (10)*	
HN3B	1.077 (3)	0.2319 (16)	0.0481 (9)	0.033 (6)*	
HN3A	0.984 (3)	0.2272 (17)	0.0012 (11)	0.046 (6)*	

### Atomic displacement parameters (Å^2^)

**Table:**

	*U*^11^	*U*^22^	*U*^33^	*U*^12^	*U*^13^	*U*^23^
C1	0.0274 (8)	0.0247 (9)	0.0245 (8)	−0.0008 (8)	−0.0019 (7)	−0.0009 (7)
C2	0.0286 (8)	0.0303 (10)	0.0254 (8)	−0.0058 (8)	−0.0033 (7)	0.0066 (7)
C3	0.0407 (10)	0.0347 (11)	0.0358 (10)	−0.0087 (9)	−0.0072 (8)	0.0085 (8)
C4	0.0443 (11)	0.0470 (14)	0.0499 (12)	−0.0198 (11)	−0.0082 (10)	0.0180 (11)
C5	0.0300 (9)	0.0640 (16)	0.0456 (12)	−0.0128 (11)	0.0021 (8)	0.0228 (12)
C6	0.0299 (9)	0.0547 (14)	0.0382 (11)	−0.0004 (10)	0.0050 (8)	0.0111 (10)
C7	0.0259 (8)	0.0376 (11)	0.0242 (9)	−0.0010 (8)	−0.0007 (7)	0.0052 (8)
C8	0.0257 (8)	0.0340 (10)	0.0231 (8)	0.0020 (8)	0.0004 (6)	0.0010 (7)
C9	0.0256 (8)	0.0243 (9)	0.0235 (8)	−0.0044 (7)	0.0028 (6)	0.0002 (7)
C10	0.0239 (7)	0.0330 (10)	0.0279 (8)	−0.0048 (8)	−0.0010 (7)	0.0047 (8)
C11	0.0274 (8)	0.0323 (10)	0.0303 (9)	−0.0040 (8)	−0.0033 (7)	0.0067 (8)
C12	0.0496 (11)	0.0329 (11)	0.0320 (10)	−0.0066 (9)	−0.0117 (9)	0.0063 (8)
C13	0.0405 (9)	0.0386 (12)	0.0305 (9)	−0.0040 (9)	−0.0032 (8)	0.0047 (8)
C14	0.0525 (11)	0.0517 (14)	0.0291 (10)	−0.0029 (11)	−0.0046 (9)	0.0090 (10)
C15	0.0509 (12)	0.0486 (14)	0.0397 (11)	−0.0048 (11)	−0.0007 (9)	0.0212 (10)
C16	0.0394 (10)	0.0349 (12)	0.0479 (12)	−0.0061 (9)	−0.0029 (9)	0.0128 (9)
C17	0.0296 (8)	0.0345 (11)	0.0345 (10)	−0.0036 (8)	−0.0009 (7)	0.0025 (8)
C18	0.0266 (8)	0.0331 (11)	0.0293 (9)	−0.0016 (8)	−0.0010 (7)	0.0037 (8)
C19	0.0260 (8)	0.0242 (9)	0.0251 (8)	0.0023 (7)	−0.0001 (6)	0.0000 (7)
C20	0.0339 (9)	0.0391 (12)	0.0355 (10)	0.0120 (9)	−0.0027 (8)	−0.0033 (9)
C21	0.0586 (13)	0.0366 (13)	0.0445 (12)	0.0161 (11)	−0.0031 (10)	−0.0029 (10)
C22	0.0613 (13)	0.0301 (11)	0.0465 (12)	−0.0004 (11)	0.0006 (10)	0.0005 (9)
C23	0.0467 (11)	0.0334 (12)	0.0450 (12)	−0.0015 (10)	0.0041 (9)	0.0059 (9)
C24	0.0297 (8)	0.0311 (11)	0.0368 (10)	0.0033 (8)	0.0038 (7)	0.0022 (8)
C25	0.0309 (9)	0.0303 (10)	0.0219 (8)	0.0057 (8)	−0.0011 (7)	0.0008 (7)
C26	0.0237 (7)	0.0289 (9)	0.0268 (8)	0.0011 (7)	−0.0005 (6)	−0.0033 (7)
C27	0.0409 (10)	0.0352 (11)	0.0290 (9)	−0.0018 (9)	−0.0006 (8)	0.0002 (8)
C28	0.0516 (12)	0.0326 (11)	0.0448 (11)	−0.0035 (10)	−0.0019 (9)	−0.0012 (9)
C29	0.0456 (10)	0.0364 (12)	0.0452 (11)	−0.0059 (10)	0.0020 (9)	−0.0125 (10)
C30	0.0449 (10)	0.0424 (12)	0.0355 (11)	−0.0055 (10)	0.0090 (9)	−0.0106 (9)
C31	0.0295 (8)	0.0382 (11)	0.0274 (9)	−0.0010 (8)	0.0012 (7)	−0.0028 (8)
N1	0.0267 (7)	0.0236 (8)	0.0251 (7)	−0.0033 (6)	0.0035 (6)	−0.0021 (6)
N2	0.0647 (11)	0.0360 (10)	0.0280 (8)	−0.0077 (9)	−0.0123 (8)	0.0022 (7)
N3	0.0224 (7)	0.0340 (9)	0.0244 (8)	0.0040 (6)	−0.0023 (6)	−0.0013 (6)
O1	0.0384 (6)	0.0295 (7)	0.0346 (7)	−0.0011 (6)	0.0070 (6)	−0.0068 (6)
O2	0.0414 (7)	0.0334 (8)	0.0374 (7)	0.0041 (6)	0.0071 (6)	−0.0081 (6)
O3	0.0316 (6)	0.0428 (8)	0.0247 (6)	−0.0066 (6)	0.0013 (5)	0.0054 (6)
O4	0.0276 (6)	0.0523 (9)	0.0349 (7)	−0.0029 (6)	−0.0065 (5)	0.0039 (6)
O5	0.0860 (13)	0.0317 (9)	0.0442 (10)	0.0013 (9)	0.0080 (9)	0.0005 (7)

### Geometric parameters (Å, °)

**Table:**

C1—O1	1.213 (2)	C23—H23B	0.9700
C1—N1	1.384 (2)	C24—C25	1.519 (3)
C1—C2	1.492 (3)	C24—H24A	0.9700
C2—C3	1.374 (3)	C24—H24B	0.9700
C2—C7	1.392 (3)	C25—N3	1.499 (3)
C3—C4	1.397 (3)	C25—H25	0.9800
C3—H3	0.9300	C26—N3	1.500 (2)
C4—C5	1.383 (4)	C26—C27	1.517 (3)
C4—H4	0.9300	C26—C31	1.527 (2)
C5—C6	1.387 (4)	C26—H26	0.9800
C5—H5	0.9300	C27—C28	1.524 (3)
C6—C7	1.385 (3)	C27—H27A	0.9700
C6—H6	0.9300	C27—H27B	0.9700
C7—C8	1.481 (3)	C28—C29	1.522 (3)
C8—O2	1.209 (2)	C28—H28A	0.9700
C8—N1	1.400 (2)	C28—H28B	0.9700
C9—N1	1.457 (2)	C29—C30	1.516 (3)
C9—C10	1.538 (2)	C29—H29A	0.9700
C9—C19	1.547 (2)	C29—H29B	0.9700
C9—H9	0.9800	C30—C31	1.522 (3)
C10—C11	1.499 (3)	C30—H30A	0.9700
C10—H10A	0.9700	C30—H30B	0.9700
C10—H10B	0.9700	C31—H31A	0.9700
C11—C12	1.363 (3)	C31—H31B	0.9700
C11—C18	1.442 (3)	N2—H2	0.8600
C12—N2	1.376 (3)	N3—HN3B	0.86 (2)
C12—H12	0.9300	N3—HN3A	0.99 (3)
C13—N2	1.373 (3)	O5—H5B	0.82 (4)
C13—C14	1.397 (3)	O5—H5C	0.89 (4)
C13—C18	1.410 (3)	O99—C88	1.145 (5)
C14—C15	1.378 (4)	N99—C88	1.289 (5)
C14—H14	0.9300	N99—C96	1.387 (7)
C15—C16	1.397 (3)	N99—C97	1.561 (6)
C15—H15	0.9300	C88—H88	0.9300
C16—C17	1.381 (3)	C96—H96A	0.9600
C16—H16	0.9300	C96—H96B	0.9600
C17—C18	1.396 (3)	C96—H96C	0.9600
C17—H17	0.9300	C97—H97A	0.9600
C19—O3	1.246 (2)	C97—H97B	0.9600
C19—O4	1.247 (2)	C97—H97C	0.9600
C20—C25	1.527 (3)	O99B—C88B	1.266 (12)
C20—C21	1.527 (3)	N99B—C88B	1.189 (13)
C20—H20A	0.9700	N99B—C97B	1.350 (18)
C20—H20B	0.9700	N99B—C96B	1.640 (12)
C21—C22	1.517 (3)	C88B—H88B	0.9300
C21—H21A	0.9700	C96B—H96D	0.9600
C21—H21B	0.9700	C96B—H96E	0.9600
C22—C23	1.521 (3)	C96B—H96F	0.9600
C22—H22A	0.9700	C97B—H97D	0.9600
C22—H22B	0.9700	C97B—H97E	0.9600
C23—C24	1.521 (3)	C97B—H97F	0.9600
C23—H23A	0.9700		
			
O1—C1—N1	124.88 (17)	H24A—C24—H24B	108.0
O1—C1—C2	128.72 (18)	N3—C25—C24	110.73 (15)
N1—C1—C2	106.39 (15)	N3—C25—C20	107.89 (15)
C3—C2—C7	121.81 (17)	C24—C25—C20	111.34 (16)
C3—C2—C1	130.83 (19)	N3—C25—H25	108.9
C7—C2—C1	107.35 (16)	C24—C25—H25	108.9
C2—C3—C4	116.9 (2)	C20—C25—H25	108.9
C2—C3—H3	121.5	N3—C26—C27	108.74 (14)
C4—C3—H3	121.5	N3—C26—C31	110.93 (15)
C5—C4—C3	121.5 (2)	C27—C26—C31	110.66 (16)
C5—C4—H4	119.2	N3—C26—H26	108.8
C3—C4—H4	119.2	C27—C26—H26	108.8
C4—C5—C6	121.27 (19)	C31—C26—H26	108.8
C4—C5—H5	119.4	C26—C27—C28	111.49 (16)
C6—C5—H5	119.4	C26—C27—H27A	109.3
C7—C6—C5	117.3 (2)	C28—C27—H27A	109.3
C7—C6—H6	121.3	C26—C27—H27B	109.3
C5—C6—H6	121.3	C28—C27—H27B	109.3
C6—C7—C2	121.15 (19)	H27A—C27—H27B	108.0
C6—C7—C8	130.40 (19)	C29—C28—C27	111.16 (18)
C2—C7—C8	108.46 (15)	C29—C28—H28A	109.4
O2—C8—N1	124.68 (17)	C27—C28—H28A	109.4
O2—C8—C7	129.50 (17)	C29—C28—H28B	109.4
N1—C8—C7	105.82 (16)	C27—C28—H28B	109.4
N1—C9—C10	111.75 (15)	H28A—C28—H28B	108.0
N1—C9—C19	111.15 (14)	C30—C29—C28	110.23 (18)
C10—C9—C19	113.34 (13)	C30—C29—H29A	109.6
N1—C9—H9	106.7	C28—C29—H29A	109.6
C10—C9—H9	106.7	C30—C29—H29B	109.6
C19—C9—H9	106.7	C28—C29—H29B	109.6
C11—C10—C9	113.96 (14)	H29A—C29—H29B	108.1
C11—C10—H10A	108.8	C29—C30—C31	112.23 (16)
C9—C10—H10A	108.8	C29—C30—H30A	109.2
C11—C10—H10B	108.8	C31—C30—H30A	109.2
C9—C10—H10B	108.8	C29—C30—H30B	109.2
H10A—C10—H10B	107.7	C31—C30—H30B	109.2
C12—C11—C18	105.80 (16)	H30A—C30—H30B	107.9
C12—C11—C10	126.61 (18)	C30—C31—C26	109.94 (16)
C18—C11—C10	127.58 (18)	C30—C31—H31A	109.7
C11—C12—N2	111.09 (18)	C26—C31—H31A	109.7
C11—C12—H12	124.5	C30—C31—H31B	109.7
N2—C12—H12	124.5	C26—C31—H31B	109.7
N2—C13—C14	129.6 (2)	H31A—C31—H31B	108.2
N2—C13—C18	108.08 (17)	C1—N1—C8	111.92 (15)
C14—C13—C18	122.2 (2)	C1—N1—C9	124.30 (14)
C15—C14—C13	117.5 (2)	C8—N1—C9	123.65 (15)
C15—C14—H14	121.3	C13—N2—C12	108.05 (17)
C13—C14—H14	121.3	C13—N2—H2	126.0
C14—C15—C16	121.1 (2)	C12—N2—H2	126.0
C14—C15—H15	119.4	C25—N3—C26	117.94 (14)
C16—C15—H15	119.4	C25—N3—HN3B	109.4 (16)
C17—C16—C15	121.4 (2)	C26—N3—HN3B	108.4 (16)
C17—C16—H16	119.3	C25—N3—HN3A	107.8 (15)
C15—C16—H16	119.3	C26—N3—HN3A	110.9 (15)
C16—C17—C18	118.99 (19)	HN3B—N3—HN3A	101 (2)
C16—C17—H17	120.5	H5B—O5—H5C	103 (3)
C18—C17—H17	120.5	C88—N99—C96	131.1 (4)
C17—C18—C13	118.79 (18)	C88—N99—C97	114.1 (4)
C17—C18—C11	134.22 (18)	C96—N99—C97	114.8 (4)
C13—C18—C11	106.97 (17)	O99—C88—N99	132.4 (4)
O3—C19—O4	125.87 (17)	O99—C88—H88	113.8
O3—C19—C9	116.25 (15)	N99—C88—H88	113.8
O4—C19—C9	117.84 (15)	N99—C96—H96A	109.5
C25—C20—C21	112.50 (17)	N99—C96—H96B	109.5
C25—C20—H20A	109.1	H96A—C96—H96B	109.5
C21—C20—H20A	109.1	N99—C96—H96C	109.5
C25—C20—H20B	109.1	H96A—C96—H96C	109.5
C21—C20—H20B	109.1	H96B—C96—H96C	109.5
H20A—C20—H20B	107.8	N99—C97—H97A	109.5
C22—C21—C20	111.41 (18)	N99—C97—H97B	109.5
C22—C21—H21A	109.3	H97A—C97—H97B	109.5
C20—C21—H21A	109.3	N99—C97—H97C	109.5
C22—C21—H21B	109.3	H97A—C97—H97C	109.5
C20—C21—H21B	109.3	H97B—C97—H97C	109.5
H21A—C21—H21B	108.0	C88B—N99B—C97B	146.5 (11)
C21—C22—C23	110.53 (19)	C88B—N99B—C96B	108.5 (8)
C21—C22—H22A	109.5	C97B—N99B—C96B	101.6 (10)
C23—C22—H22A	109.5	N99B—C88B—O99B	131.1 (10)
C21—C22—H22B	109.5	N99B—C88B—H88B	114.5
C23—C22—H22B	109.5	O99B—C88B—H88B	114.5
H22A—C22—H22B	108.1	N99B—C96B—H96D	109.5
C24—C23—C22	110.70 (17)	N99B—C96B—H96E	109.5
C24—C23—H23A	109.5	H96D—C96B—H96E	109.5
C22—C23—H23A	109.5	N99B—C96B—H96F	109.5
C24—C23—H23B	109.5	H96D—C96B—H96F	109.5
C22—C23—H23B	109.5	H96E—C96B—H96F	109.5
H23A—C23—H23B	108.1	N99B—C97B—H97D	109.5
C25—C24—C23	111.33 (16)	N99B—C97B—H97E	109.5
C25—C24—H24A	109.4	H97D—C97B—H97E	109.5
C23—C24—H24A	109.4	N99B—C97B—H97F	109.5
C25—C24—H24B	109.4	H97D—C97B—H97F	109.5
C23—C24—H24B	109.4	H97E—C97B—H97F	109.5

### Hydrogen-bond geometry (Å, °)

**Table:**

*D*—H···*A*	*D*—H	H···*A*	*D*···*A*	*D*—H···*A*
N3—HN3B···O4^i^	0.86 (3)	1.88 (3)	2.7309 (19)	169 (2)
N2—H2···O5^ii^	0.86	1.97	2.813 (3)	165
N3—HN3A···O3^iii^	0.99 (3)	1.79 (3)	2.7740 (19)	173 (2)
O5—H5B···O99	0.83 (4)	1.84 (4)	2.645 (4)	164 (4)
O5—H5C···O1^iv^	0.89 (4)	1.99 (4)	2.857 (2)	164 (3)

Symmetry codes:  (i) *x*+1, *y*, *z*; (ii) *x*+1/2, −*y*+1/2, −*z*+1; (iii) *x*+1/2, −*y*+1/2, −*z*; (iv) −*x*+1/2, −*y*, *z*+1/2.
